# Breeding Increases the Efficacy of *Chondrostereum purpureum* in the Sprout Control of Birch

**DOI:** 10.1371/journal.pone.0117381

**Published:** 2015-02-12

**Authors:** Leena Hamberg, Henna Vartiamäki, Jarkko Hantula

**Affiliations:** Finnish Forest Research Institute, Vantaa Research Unit, Vantaa, Finland; Georg-August-University Göttingen, GERMANY

## Abstract

We tested whether the pairing of selected isolates could be used to increase the efficiency of a decay fungus *Chondrostereum purpureum* (Pers. Ex Fr.) Pouzar to control hardwood sprouting in Finland. We paired *C. purpureum* strains efficient in sprout control or highly active in laccase production, and tested the efficacy of their progeny in spout control experiments. This procedure resulted in a strain with an efficacy superior to that of the parental strains. The mortality of birch (*Betula pendula* Roth. and *B. pubescens* Ehrh.) 1 cm in stump diameter was 78%, 56% and 9% for the best progeny, the best parental strain and the control, respectively. Mortality was only slightly higher for *B. pendula* than for *B. pubescens* but no significant differences were found between the number or maximum height of stump sprouts. Our results showed that cross breeding of this decay fungus is a good alternative in attempts to produce efficient biocontrol agents against hardwood sprouting.

## Introduction

In Finland, sprouting of broad-leaved trees is a hindrance in spruce (*Picea abies* [L.] H. Karst.) and pine (*Pinus sylvestris* L.) regeneration areas, alongside roads and railways, under electric power lines and above gas pipe lines. In regeneration areas, broad-leaved species such as silver and downy birch (*Betula pendula* Roth. and *B*. *pubescens* Ehrh.), decrease the growth of more commercially valuable conifers, and therefore non-crop species are typically cleaned, preferably at an early stage when trees are about 1 m in height [1,2,3]. Next to roads and railways, broad-leaved trees form a threat to the safety of traffic as they restrict visibility, cover traffic signs and tempt moose, and are therefore regularly removed. Electric power lines are kept open to ensure continuous electric transmission and gas pipe lines marked with visible signs are frequently cleared in order to avoid unintended excavations. Sprout control of broad-leaved trees costs more than 60 million euros annually in Finnish forest regeneration areas, along roads and railways, and at electric power and gas pipe lines (information gathered from UPM Forest Ltd., The Finnish Transport Agency, Fingrid Ltd. and Gasum Ltd.).

Herbicides were previously successfully used in sprout control [4,5], but the use of chemicals is no longer recommended in Finnish groundwater areas due to their harmful effects [6,7]. Also, public opinion is strongly against their usage. On the other hand, mechanical cutting alone is not effective due to the vigorous sprouting of broad-leaved trees [8,9]. Therefore, new methods for sprout control are needed.

One promising option is to utilize a natural pathogen of broad-leaved trees, the silver leaf fungus, *Chondrostereum purpureum* (Pers. ex Fr.) Pouzar [10,11], which has been shown to restrict the sprouting of several tree species [8,12,13,14]. Therefore it can be considered a ‘nature friendly’ alternative to chemicals.

*C*. *purpureum* is a basidiomycete commonly found on wounded broad-leaved wood in boreal and temperate vegetation zones in Europe [15,16,17]. The fungus is widespread in nature due to its efficient basidiospore production resulting in frequent new infections [15,18]. In nature, monokaryotic spores landing on wood first germinate, after which the hyphae from different spores hybridize and form dikaryotic mycelia which then colonize the host [18]. However, in man-made sprout control, dikaryotic fungal hyphae are directly spread onto freshly cut stumps [9,12,13].

The use of *C*. *purpureum* in sprout control is based on its ability to grow inside a stump and finally decay it. During the invasion, *C*. *purpureum* penetrates through starch granules and cell walls enzymatically, and induces the occlusion of tree vessels [15,19,20,21]. The resulting dehydration combined with fungal toxins may finally cause mortality of the host. White rot fungi, such as *C*. *purpureum*, are efficient in breaking down lignin [22,23]. In woody cell walls, lignin surrounds the cellulose which is the actual carbon and energy source for the fungus, and possibly the ultimate reason for lignin degradation [23,24]. Laccases are the main oxidative enzymes in this decay process in addition to lignin and manganese peroxidases [13,23].

The efficacy of *C*. *purpureum* in sprout control depends on the host tree species [5,8,9,13,14]. Also, an increase in the diameter of an inoculated stump has been shown to affect stump mortality [9,14]. Furthermore, considerable variation exists in the ability of different *C*. *purpureum* strains to prevent sprouting [4,5,13], but the possibility of increasing control efficiency by breeding has not been tested before.

Breeding among sexually propagating fungi has previously been used in industrial applications such as the chemical industry (e.g. enzymes for bioethanol), and in wine production in order to increase the yield of cultivated organisms and economic benefit [25,26]. Moreover, the biocontrol ability of a saprophytic fungus *Phlebiopsis gigantea* (Fr.) Jül. against a root rot fungus *Heterobasidion parviporum* Niemelä & Korhonen was improved by traditional breeding [27]. Therefore, breeding based on the natural variation of *C*. *purpureum* can be expected to provide an efficient way to increase the ability of this fungus to control sprouting.

The main aim of this study was to test whether breeding can be used to increase the efficacy of the decay fungus *C*. *purpureum* in preventing sprouting of small birch stumps (*Betula pendula* and *B*. *pubescens*). We hypothesized that pairing of strains efficient in (i) sprout control and (ii) laccase production could result in a superior combination of genes producing at least one progeny strain better than the parental strains. In addition, we tested whether the ability of *C*. *purpureum* to control sprouting differs between the birch species. Here we show that breeding can successfully be used to improve the biocontrol ability of *C*. *purpureum* but no significant differences between the two birch species investigated were found.

## Materials and Methods

**Fig 1:**
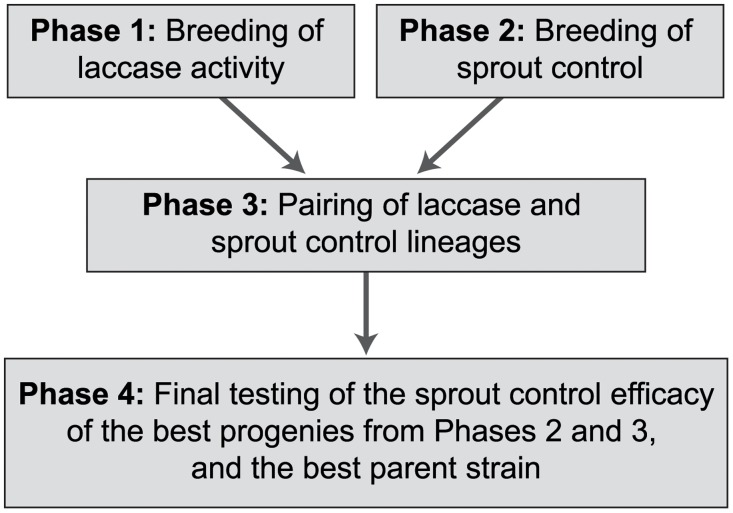
The Breeding Process of the Study.

The study was composed of four phases (Fig. 1): 1) pairing of the best strains in terms of laccase activity and progeny testing in the laboratory, 2) pairing of the most efficient parental strains available [13] and progeny testing in the field, 3) pairing of the best progenies from Phases 1 and 2 and progeny testing in the field, and 4) final testing of the efficacy of the best progenies from Phases 2 and 3. All data sets and figures are available from the Dryad Digital Repository: http://dx.doi.org/10.5061/dryad.f5s6r

### Ethics Statement

Permission for the field experiments of Phases 2–4 was granted by land owners, Fingrid Ltd., UPM-Kymmene Ltd. and the Finnish Forest Research Institute. Endangered or protected species were not used in this study. Fungal strains of *C*. *purpureum* have been deposited in the culture collection of the Finnish Forest Research Institute.

### Laccase activity tests

Laccase activity of the isolates was studied as it has been shown that laccase activity of *C*. *purpureum* isolates correlates with the efficacy of their sprout control [13]. In order to find efficient laccase producers, altogether 69 heterocaryotic *C*. *purpureum* isolates were collected from birch (*Betula pendula* and *B*. *pubescens*) stumps in July-October 2009. These isolates were collected from different parts of Finland, in Alavus (3 isolates), Vantaa (40), Hyytiälä (1), Juupajoki (2), Mäntsälä (16) and Järvenpää (8) (Table 1).

**Table 1:** *Chondrostereum purpureum* Isolates Collected for Phase 1.

No.	Isolate	Laccase activity^**a**^	Municipality/locality	Collected by	Date of isolation
1	AL1	1	Alavus, Murronneva	H. Vartiamäki	2009–07–19
2	AL2	2	Alavus, Murronneva	H. Vartiamäki	2009–07–19
3	AL3	2	Alavus, Murronneva	H. Vartiamäki	2009–07–19
4	KY1	1	Vantaa, Kylmäoja	H. Vartiamäki	2009–08–07
5	KY2	1	Vantaa, Kylmäoja	H. Vartiamäki	2009–08–07
6	KY3	3	Vantaa, Kylmäoja	H. Vartiamäki	2009–08–07
7	KY4	1	Vantaa, Kylmäoja	H. Vartiamäki	2009–08–07
8	KY5	3	Vantaa, Kylmäoja	H. Vartiamäki	2009–08–07
9	KY6	2	Vantaa, Kylmäoja	H. Vartiamäki	2009–08–07
10	KY7	2	Vantaa, Kylmäoja	H. Vartiamäki	2009–08–17
11	KY8	2	Vantaa, Kylmäoja	H. Vartiamäki	2009–08–17
12	KY9	3	Vantaa, Kylmäoja	H. Vartiamäki	2009–08–17
13	KY10	2	Vantaa, Kylmäoja	H. Vartiamäki	2009–08–17
14	KY11	3	Vantaa, Kylmäoja	H. Vartiamäki	2009–09–02
15	KY12	1	Vantaa, Kylmäoja	H. Vartiamäki	2009–09–02
16	KY13	3	Vantaa, Kylmäoja	H. Vartiamäki	2009–09–02
17	KY14	1	Vantaa, Kylmäoja	H. Vartiamäki	2009–09–02
18	KY15	2	Vantaa, Kylmäoja	H. Vartiamäki	2009–09–02
19	KY16	1	Vantaa, Kylmäoja	H. Vartiamäki	2009–09–02
20	KY17	3	Vantaa, Kylmäoja	H. Vartiamäki	2009–09–02
21	KY18	3	Vantaa, Kylmäoja	H. Vartiamäki	2009–09–02
22	KY19	3	Vantaa, Kylmäoja	H. Vartiamäki	2009–09–02
23	KY20	2	Vantaa, Kylmäoja	H. Vartiamäki	2009–09–02
24	KY21	1	Vantaa, Kylmäoja	H. Vartiamäki	2009–09–02
25	KY22	3	Vantaa, Kylmäoja	H. Vartiamäki	2009–09–02
26	KY23	3	Vantaa, Kylmäoja	H. Vartiamäki	2009–09–02
27	KY24	2	Vantaa, Kylmäoja	H. Vartiamäki	2009–09–02
28	KY25	3	Vantaa, Kylmäoja	H. Vartiamäki	2009–09–02
29	KY26	3	Vantaa, Kylmäoja	H. Vartiamäki	2009–09–02
30	KY27	2	Vantaa, Kylmäoja	H. Vartiamäki	2009–09–14
31	KY28	3	Vantaa, Kylmäoja	H. Vartiamäki	2009–09–14
32	JU1	2	Juupajoki, Hyytiälä	A. Uotila	2009–08–26
33	OR1	3	Orivesi	A. Uotila	2009–09–14
34	HA1	2	Mäntsälä, Kortistonkulma	L. Hamberg	2009–10–06
35	HA2	1	Mäntsälä, Kortistonkulma	L. Hamberg	2009–10–06
36	HA3	2	Mäntsälä, Kortistonkulma	L. Hamberg	2009–10–06
37	OH1	1	Mäntsälä, Kivistönkulma	L. Hamberg	2009–10–06
38	OH2	2	Mäntsälä, Kivistönkulma	L. Hamberg	2009–10–06
39	JÄ1	2	Järvenpää, Paavonpolku	L. Hamberg	2009–10–06
**40**	**JÄ2**	**3**	**Järvenpää, Paavonpolku**	**L. Hamberg**	**2009–10–06**
41	JÄ3	2	Järvenpää, Paavonpolku	L. Hamberg	2009–10–06
42	JÄ4	1	Järvenpää, Paavonpolku	L. Hamberg	2009–10–06
43	JÄ5	1	Järvenpää, Paavonpolku	L. Hamberg	2009–10–06
44	JÄ6	1	Järvenpää, Paavonpolku	L. Hamberg	2009–10–06
45	JÄ7	1	Järvenpää, Paavonpolku	L. Hamberg	2009–10–06
46	JÄ8	1	Järvenpää, Paavonpolku	L. Hamberg	2009–10–06
47	PI1	3	Mäntsälä, Pirjola	L. Hamberg	2009–10–11
48	PI2	2	Mäntsälä, Pirjola	L. Hamberg	2009–10–11
49	PI3	1	Mäntsälä, Pirjola	L. Hamberg	2009–10–11
50	PI4	3	Mäntsälä, Pirjola	L. Hamberg	2009–10–11
51	PI5	1	Mäntsälä, Pirjola	L. Hamberg	2009–10–11
52	PI6	1	Mäntsälä, Pirjola	L. Hamberg	2009–10–11
**53**	**PI7**	**3**	**Mäntsälä, Pirjola**	**L. Hamberg**	**2009–10–11**
54	PI8	2	Mäntsälä, Pirjola	L. Hamberg	2009–10–11
55	PI9	2	Mäntsälä, Pirjola	L. Hamberg	2009–10–11
56	PI10	1	Mäntsälä, Pirjola	L. Hamberg	2009–10–11
57	PI11	3	Mäntsälä, Pirjola	L. Hamberg	2009–10–11
58	RU1	3	Vantaa, Ruskeasanta	L. Hamberg	2009–10–19
59	RU2	3	Vantaa, Ruskeasanta	L. Hamberg	2009–10–19
60	RU3	2	Vantaa, Ruskeasanta	L. Hamberg	2009–10–19
61	RU4	3	Vantaa, Ruskeasanta	L. Hamberg	2009–10–19
62	RU5	1	Vantaa, Ruskeasanta	L. Hamberg	2009–10–19
63	RU6	2	Vantaa, Ruskeasanta	L. Hamberg	2009–10–19
64	RU7	1	Vantaa, Ruskeasanta	L. Hamberg	2009–10–19
65	RU8	2	Vantaa, Ruskeasanta	L. Hamberg	2009–10–19
66	RU9	1	Vantaa, Ruskeasanta	L. Hamberg	2009–10–19
67	RU10	2	Vantaa, Ruskeasanta	L. Hamberg	2009–10–19
68	RU11	2	Vantaa, Ruskeasanta	L. Hamberg	2009–10–19
69	RU12	1	Vantaa, Ruskeasanta	L. Hamberg	2009–10–19

All *C*. *purpureum* strains were isolated from fruiting bodies on birch stumps (*Betula pendula* and *B*. *pubescens*) in Finland and were heterokaryotic. The two best strains in the laccase activity test are in bold.

^a^ 0: No color reaction, i.e., low laccase activity; 1: slight color reaction; 2: intermediate color reaction; 3: strong color reaction, i.e., high laccase activity on Petri plates.

We used ABTS (2,2’-azino-bis[3-ethylbenzothiazoline-6-sulphonic acid]) plates whose color reaction reveals the laccase activity of *C*. *purpureum* (0 = no color reaction, i.e., low laccase activity to 3 = strong dark-green color reaction, i.e., high laccase activity, see [13]). The ABTS plates (modified from [28]) were prepared by mixing 1.0 g glucose (Acros Organics), 2.0 g KH_2_PO_4_ (Merck), 0.5 g MgS0_4_ × 7H_2_O (Fluka), 0.13 g CaCl_2_ × 2H_2_O (Merck), 0.5 g (NH_4_)_2_-tartrate (Alfa Aesar), 1.78 g dimethylsuccinic acid (Merck), 0.2 g yeast extract (Becton, Dickinson and Company) and 25 g agar (Becton, Dickinson and Company) with 1000 ml deionized water. All elements except agar were suspended in water and pH adjusted to 5.0 with 1.0 M NaOH. Agar was added and the substrate was autoclaved at 121°C for 15 min. Altogether 250 mg ABTS (Applichem) diluted with 99% ethanol was added to the medium when the temperature was 48–50°C.

All *C*. *purpureum* isolates were grown on potato dextrose agar Petri plates (PDA: 24 g potato dextrose broth and 15 g agar with 1000 ml deionized water; Becton, Dickinson and Company), and a 6-mm diameter agar plug at the periphery of the grown mycelium was transferred to the middle of an ABTS plate using a sterilized Pasteur pipette and a scalpel. Two replicate plates per isolate were incubated at 25°C for 4 d. Enzymatic activity was visually estimated as the strength of the color reaction on a plate. The two isolates with the highest laccase activity were selected and paired (see below) with each other (see Tables 1 and 2, Phase 1). Laccase activities of the 24 progeny isolates were also investigated after 4 d on the ABTS plates as described above, and the best producer was named E+_1_.

**Table 2:** Breeding Process of the Study.

Phase 1			Phase 2			Phase 3		
Progeny	Parent strain/ spore number	Laccase activity^**a**^	Progeny	Parent strain/ spore number	*p* ^**b**^	Progeny	Parent strain/ spore number	*p* ^**b**^
R2_1_	JÄ2/2 × PI7/2	3	R1_2_	3.11/1 × HY4/1	0.312 / 0.213	R1_3_	V2_2_/1 × E+_1_/1	0.474 / 0.332
R3_1_	JÄ2/3 × PI7/3	3	R2_2_	3.11/2 × HY4/2	0.282 / 0.191	R2_3_	V1_2_/2 × E+_1_/2	0.376 / 0.529
R4_1_	JÄ2/4 × PI7/4	3	R3_2_	3.11/3 × HY4/3	0.374 / 0.262	**R3**_3_	**V1**_**2**_ **/3 × E+**_**1**_ **/3**	0.323 **/** 0.214
R5_1_	JÄ2/5 × PI7/5	3	R4_2_	3.11/4 × HY4/4	0.204 / 0.134	R4_3_	V1_2_/4 × E+_1_/4	0.799 / 0.611
R6_1_	JÄ2/6 × PI7/6	3	R5_2_	3.11/5 × HY4/5	0.185 / 0.120	**R5**_3_	**V1**_**2**_ **/5 × E+**_**1**_ **/5**	0.304 **/** 0.199
**R7**_**1**_ **= E+**_**1**_	**JÄ2/7 × PI7/7**	**3**	R6_2_	3.11/6 × P4/1	0.823 / 0.990	R6_3_	V1_2_/6 × E+_1_/6	0.272 / 0.175
R8_1_	JÄ2/8 × PI7/8	2	R7_2_	3.11/7 × P4/2	0.771 / 0.600	R7_3_	V2_2_/1 × E+_1_/7	0.817 / 0.627
R9_1_	JÄ2/9 × PI7/9	2	R8_2_	3.11/8 × P4/3	0.299 / 0.204	R8_3_	V2_2_/2 × E+_1_/8	0.959 / 0.759
R10_1_	JÄ2/10 × PI7/10	3	R9_2_	3.11/9 × P4/4	0.425 / 0.302	**R9**_3_	**V2**_**2**_ **/3 × E+**_**1**_ **/9**	0.153 **/** 0.092
R11_1_	JÄ2/11 × PI7/11	2	R10_2_	3.11/1 × P4/5	0.114 / 0.070	R10_3_	V2_2_/4 × E+_1_/10	0.596 / 0.433
R12_1_	JÄ2/12 × PI7/12	3	R11_2_	HY4/6 × P4/6	0.327 / 0.225	R11_3_	V2_2_/5 × E+_1_/11	0.614 / 0.803
R14_1_	JÄ2/14 × PI7/14	3	**R12**_**2**_ **= V1**_**2**_	**HY4/7 × P4/7**	0.109 / 0.067	R12_3_	V2_2_/6 × E+_1_/12	0.198 / 0.302
R15_1_	JÄ2/15 × PI7/15	1	R13_2_	HY4/8 × P4/8	0.813 / 0.637			
R16_1_	JÄ2/16 × PI7/16	2	R14_2_	HY4/9 × P4/9	0.361 / 0.251			
R17_1_	JÄ2/17 × PI7/17	3	**R15**_**2**_ **= V2**_**2**_	**HY4/10 × P4/10**	0.071 / **0.042**			
R18_1_	JÄ2/18 × PI7/18	1						
R19_1_	JÄ2/19 × PI7/19	3						
R20_1_	JÄ2/20 × PI7/20	3						
R21_1_	JÄ2/21 × PI7/21	3						
R22_1_	JÄ2/22 × PI7/1	3						
R24_1_	JÄ2/24 × PI7/3	1						
R25_1_	JÄ2/25 × PI7/4	3						
R26_1_	JÄ2/26 × PI7/5	2						
R27_1_	JÄ2/27 × PI7/7	3						

**Fig 2:**
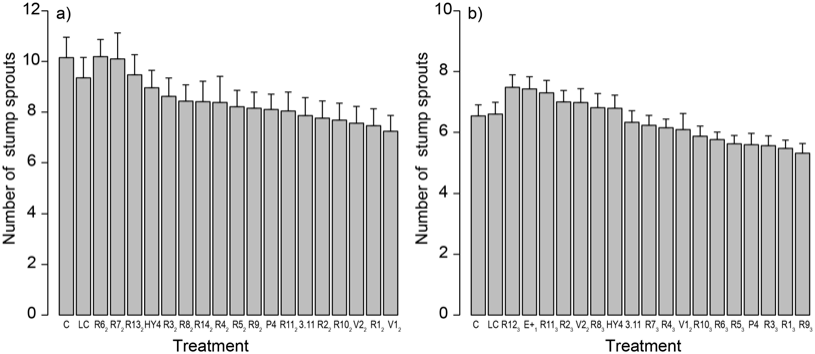
The Effects of Different Treatments on Birch Spouting during Phases 2 and 3. The number of stump sprouts of birch (*Betula pendula* and *B*. *pubescens*) per investigated stump one growing season after the treatments a) in Phase 2 (Porvoo in 2009) and b) Phase 3 (Turenki in 2010). C = control, i.e., cutting only; LC = liquid control, i.e., cut stumps were spread with the inoculum medium without *C*. *purpureum*; HY4, P4 and 3.11 = the best parental strains of *C*. *purpureum*; E+_1_, R1_2_-R11_2_, R13_2_-R14_2_, V1_2_-V2_2_, and R1_3_-R12_3_ = progenies of *C*. *purpureum* strains, see Table 2. Means with standard errors are presented.

Breeding phase, *C*. *purpureum* strain and its spore number in each breeding phase are presented. The best progenies in terms of laccase activity (the strongest color reaction, Phase 1) or in terms of efficacy in sprout control in the field (Phases 2 and 3) three months after *C*. *purpureum* application are in bold (see Fig. 2). During Phase 2 the best parent strains based on field experiments were bred and in Phase 3 the best progenies from Phases 1 and 2 were bred. Note: the subscript of a progeny indicates the breeding phase.

^a^ 0: No color reaction, i.e., low laccase activity; 1: slight color reaction; 2: intermediate color reaction; 3: strong color reaction, i.e., high laccase activity on a Petri plate.

^b^ Statistical difference in the number of stump sprouts between the control (cutting only) and the *C*. *purpureum* treatment, and the liquid control (inoculum medium spread without *C*. *purpureum*) and the *C*. *purpureum* treatment, respectively. *P*-values indicating statistically significant differences are based on generalized linear mixed models. *P*-values < 0.05 are in bold and those between 0.05 and 0.10 have been underlined.

### Pairings

First, the best two *C*. *purpureum* strains in terms of laccase activity (see Table 1) were paired (Table 2, Phase 1). The isolates to be paired were cultured on PDA Petri plates, one isolate per plate, for ca. 3–4 weeks until they formed fruiting bodies. A fruiting body was cut from a plate with a sterilized scalpel and transferred onto a new PDA plate lid. One drop of sterilized water was pipetted just next to the fruiting body to activate spore release. One day later, when spores had started to germinate on a PDA Petri plate, 24 spores per isolate JÄ2 and 20 spores per isolate PI7 were successfully isolated from the plates with a modified and sterilized Pasteur pipette and transferred to new PDA plates, one spore per plate. After the occurrence of hyphae their morphology was investigated with a microscope to verify that single spore isolations were successful and that the hypha was homokaryotic (no visible clump connections). A single hyphal tip was further isolated from each plate to verify a homokaryotic state. These isolates were paired according to Table 2 (Phase 1), i.e., two spores from different isolates were placed on a single PDA Petri plate to allow them to form heterokaryotic mycelium. After one week, an interaction zone between the isolates developed between the inocula, from which hyphae were transferred to a new PDA Petri plate. Successful pairing was verified microscopically by confirming clump connections. A single heterokaryotic hyphal tip was further transferred to a new PDA plate for further use.

The three most efficient natural strains tested by Vartiamäki et al. [13], isolates HY4, P4 and 3.11, were originally collected from birches (*Betula pendula* and *B*. *pubescens*) in Juupajoki in 2003, Vantaa in 2004 and Helsinki in 2001, respectively, and used for the second set of pairings. Altogether 10 spores per isolate HY4 and P4, and 9 spores per isolate 3.11 were successfully isolated from the plates and paired as described above (see Table 2, Phase 2).

Third, the best progenies from Phases 1 and 2 were paired according to Table 2 (Phase 3), as described above.

### Inoculum for field experiments

The inoculum medium for the field experiments was prepared as follows: 24 g potato dextrose broth (Becton, Dickinson and Company) and 20 g Sipernat 22S (Evonik Degussa) per 1000 ml deionized water was autoclaved in an Agarmatik machine at 121°C for 15 min. Erlenmayer flasks, 250 ml in volume were also autoclaved at 121°C for 15 min. Altogether, 150 ml cooled autoclaved inoculum medium was added to the flasks and *C*. *purpureum* hyphae from one PDA cellophane plate (per isolate, Phase 2) was transferred to the flask with a sterilized scalpel. This inoculated medium was incubated in the dark at 18°C for 7–10 d on a rotator shaker (100 rpm). The inoculum was homogenized by Ultra Turrax apparatus for 1.5 min and diluted 1:10 with tap water before treatments in the field.

For Phases 3 and 4, the fungal inoculum was prepared as in Phase 2 except that the weight of hyphae added to the Erlenmayer bottles was equal (0.120 ± 0.024 g and 0.167 ± 0.018 g, mean ± SD, respectively) in each treatment (i.e., for each fungal isolate used in the experiment). All strains were cultivated for 10 d in a shaker at 20°C.

### Experiments in the field

The efficacy of all progenies of Phases 2, 3 and 4 was investigated in field experiments. The first experiment (see Table 2, Phase 2) was established on 9 and 10 June 2009 under electric power lines in Porvoo, Hinthaara, southern Finland. Both days were cloudy with a temperature of ca. 15°C. However, 9 June was partly drizzly. The site included plenty of naturally growing birches (*Betula pendula* and *B*. *pubescens*) with a basal diameter (at ca. 15 cm above soil surface) of 2.2 ± 1.4 cm (mean ± SD). The efficacy of 15 different progenies and their parental strains HY4, 3.11 and P4 were tested in this field experiment (see Table 2). Furthermore, sample plots for controls (cutting only) and liquid controls (procedural controls: inoculum medium without *C*. *purpureum*) were also established. Altogether, 80 circular sample plots, four per isolate or control treatment, were established randomly on the site. Each sample plot included ca. 20 birch stumps. Thus, altogether 1583 birch stumps were investigated in this experiment (*Betula bendula* 49%, *B*. *pubescens* 49% and unrecognized birches 2%—the proportion of tree species was investigated three months after the treatments and therefore some were already dead). In the *C*. *purpureum* sample plots, fungal inoculum was spread on stumps immediately after cutting. Treatments in the liquid control sample plots were similar, but only inoculum medium without any mycelium was sprayed on the stumps. Viability of the fungal inoculum was confirmed before and after field applications by squirting inoculum to PDA Petri plates. All of the *C*. *purpureum* isolates were viable before and after the experiment.

Three and a half months later, after one growing season, in September 2009, the number of living sprouts per birch stump and basal diameters of stumps (mm) were measured. Based on the number of sprouts, the two best *C*. *purpureum* progenies were chosen for further investigation. The two best ones had the lowest number of stump sprouts per stump and the statistical difference was the highest (although usually not significant) when compared to the control stumps. These progenies were named V1_2_ and V2_2_ (see Table 2).

In the second field experiment, the efficacy of the progenies and their parental strains in Phase 3 (Table 2) were tested in the field. The efficacy of different *C*. *purpureum* strains was tested in three different regeneration areas of spruce in Turenki, southern Finland, including lots of naturally grown birches (*Betula pendula* and *B*. *pubescens*) 1.0 ± 0.4 cm (mean ± SD) in basal diameter (at ca. 15 cm above soil surface). At each site, the efficacy of 12 progenies and the parental strains from different phases, i.e., HY4, P4, 3.11 and V1_2_, V2_2_, E+_1_, was tested. Furthermore, sample plots for controls (cutting only) and liquid controls (inoculum medium spread without *C*. *purpureum*) were established. One sample plot per treatment was randomly placed within each site. Each sample plot included ca. 30 birch stumps. Thus, 60 sample plots including 1796 birch stumps were included in the study (*Betula pendula* 46%, *B*. *pubescens* 53%, and unrecognized birches 1%). Sample plots were treated on 15 and 16 June 2010. 15 June was partly cloudy, sunny and rainy with 15°C whereas 16 June was sunny but windy with 17°C. All *C*. *purpureum* strains were viable before and after the experiment.

Three months later, in September 2010, the number of stump sprouts per birch stump and basal diameters of stumps (mm) were measured, and the three best *C*. *purpureum* progenies were chosen for further investigation. The three best strains had the lowest numbers of stump sprouts and statistical differences were the highest (although usually not significant) when compared to the control stumps.

In the third field experiment we tested the efficacy of the best *C*. *purpureum* strains from Phases 2 and 3 for three growing seasons. The best strains from Phase 3, R3_3_ (progeny strain from the pairing between V1_2_ and E+_1_), R5_3_ (V1_2_ × E+_1_) and R9_3_ (V2_2_ × E+_1_), together with strains V1_2_ and V2_2_ from Phase 2 were included in the experiment. Furthermore, HY4, the best parental strain originally collected from the field (based on the study by Vartiamäki et al. [13]), and a liquid control (inoculum medium without *C*. *purpureum*) were included. Eight regeneration areas of spruce with a high frequency of birches (*Betula pendula* and *B*. *pubescens*) were chosen for the study (Table 3). Four of the sites were located in Lapinjärvi, and four in Turenki, both in southern Finland. At each site, circular sample plots including ten birch saplings per treatment were established. The order of the sample plots was randomized within each site. Altogether 560 birch saplings, 80 saplings per treatment, were included in the study (*Betula pendula* 12%, *B*. *pubescens* 75% and unrecognized birches 13%). The mean basal diameter of birches was 1.1 ± 0.4 cm (mean ± SD, ca. 15 cm above soil surface). The experiment was established on 10 to 13 May 2011. The weather was sunny with a temperature of 14–21°C.

**Table 3:** Description of the Study Sites Included in the Field Experiment in Phase 4.

Site	Site type^**a**^	Topography	Soil	Clear-cutting (year)	Soil cultivation	Spruce saplings planted (year)
Lapinjärvi 67	MT	Partly sloping	Sand moraine	2003	Mounding	2004
Lapinjärvi 58	MT	Even	Gravel moraine/stony	1998	No	1999
Lapinjärvi 245	OMT	Even	Clay/gravel moraine	1999	Disc trenching	2000
Lapinjärvi 349	OMT	Even	Clay/gravel moraine/peat-covered	2006^b^	Disc trenching	2007
Turenki 93	OMT	Even	Gravel moraine	2002	Mounding	2003
Turenki 116	OMT	Even	Gravel moraine	2004	No	2004
Turenki 220	OMT	Even	Gravel moraine	2003	Mounding	2004/2007
Turenki 241.2	OMT	Even	Peat	2003	Mounding	2004–2005

^a^ MT = *Myrtillus* type forest, OMT = *Oxalis*—*Myrtillus* type forest.

^b^ Windfall spruces on this site.

The mortality of stumps, the number and maximum height of stump sprouts in living stumps and the occurrence of fruiting bodies were investigated one, two and three growing seasons (2011, 2012 and 2013) after the treatments. Furthermore, the basal area of stumps was measured (mm), and the number of other saplings (cut or uncut), and retention trees (i.e., mature trees left on sites, diameter at breast height ≥ 5 cm, m^3^ ha^-1^) around an investigated stump were measured as these have an effect on sprouting [9]. The number of other stumps and saplings around an investigated sapling was measured within a circular subsample plot 0.5 m in radius whereas the diameters of retention trees (cm) at breast height were measured within a circular sample plot 10 m in radius. Tree volume was calculated using the models by Laasasenaho [29]. The occurrence of moose browsing was recorded per investigated stump to take this into account as it has an effect on the height of stump sprouts.

### Statistical analyses

Generalized linear mixed models (GLMMs) were used to investigate differences between the treatments. In Phases 2 and 3 the effects of different treatments (control, liquid control and different *C*. *purpureum* strains) on the number of stump sprouts were investigated using a Poisson model with log link function in package lme4 in the statistical program R [30,31]. Thus, the number of stump sprouts per stump three months after the treatments was the response variable and treatments (as a factor) and the basal diameter of a stump were explanatory variables in the models. In Phase 2, the sample plot was used as a random factor to take into account pseudoreplication, i.e., the fact that 20 stumps within each sample plot may be—due to environmental conditions—more similar than randomly chosen saplings. In Phase 3, site (logging unit) and sample plot were treated as nested random factors to take into account the fact that several stumps within the same site and sample plot were investigated. Both in Phase 2 and 3, the control treatment (cutting only) and the liquid control (inoculum medium without a fungus) were compared to the other treatments. The best fungal strains were chosen for further investigation based on the biggest differences in the number of stump sprouts per stump between the control treatments and the fungal treatments (lower in the fungal treatment) and the lowest *p*-values.

The GLMMs were also used to analyze data relating to the final field experiment, which lasted for three growing seasons from 2011 to 2013 (Phase 4). We estimated the models separately for each year. The package lme4 in R was used to investigate the effects of controls (inoculum without *C*. *purpureum* spread on cut stumps) and different *C*. *purpureum* strains on the investigated stumps. Effects on the mortality of investigated stumps and the probability of occurrence of fruiting bodies on stumps were analyzed using a binomial model with logit link function [30,31]. Effects on the number of stump sprouts per stump were investigated using a Poisson model with log link function. The effect of different treatments on the maximum height of stump sprouts per stump was investigated using function lme in the nlme package in R [31,32] assuming a normal distribution. All stumps were included in the mortality models whereas only living stumps were included when differences between the number and maximum height of stump sprouts were investigated. Explanatory variables in the models were 1) treatment (as a factor with seven levels; however, the liquid control was excluded from the fruiting body model), 2) the basal diameter of a stump (mm), 3) the number of other stumps and saplings around an investigated stump, and 4) the total volume of retention trees around an investigated sapling (m^3^ ha^-1^). For the stump sprout height model 5) browsing (as a factor: 0 = no browsing, 1 = browsing) was also taken into account as it affects the maximum height of stump sprouts. First, differences between the liquid control and the *C*. *purpureum* treatments were investigated. Second, differences between the best fungus treatment (the greatest difference from the control) and other *C*. *purpureum* treatments were investigated. Correlations between the continuous explanatory variables were low (the strongest Pearson correlation was between the basal diameter of a stump and the total volume of retention trees, *r* = 0.14), and therefore all of the explanatory variables were included in the models. Forest site and sample plot were used as nested random factors in the models.

Differences in mortality, sprout number and maximum height between birch species (*Betula pendula* and *B*. *pubescens*) were investigated as above. However, tree species (as a factor with two levels: *B*. *pendula* and *B*. *pubescens*) was included as an explanatory variable to the models instead of treatment. The data collected in 2013 including sample plots treated with *C*. *purpureum* strains were used in these investigations. Unrecognized dead stumps were removed from the mortality model.

## Results

### Phase 1: Laccase activity tests and pairings

Altogether 69 *C*. *purpureum* strains were collected from birch stumps, and investigated on ABTS Petri plates revealing laccase activity. Based on the color reaction on these plates, ca. 32% of the investigated strains belonged to class 3 (strong color reaction, i.e., high laccase activity, Table 1, see [13]). The best *C*. *purpureum* strains in this enzymatic test were JÄ2 and PI7 (the strongest color reaction on plates), which were further paired with each other (Table 2, Phase 1). The laccase activity of the progenies of JÄ2 × PI7 was further tested on the ABTS plates. Altogether 67% of the strains belonged to class 3 (strong color reaction, i.e., high laccase activity, Table 2). The best of the progenies in this enzymatic test (showing the strongest color reaction on plates) was R7_1_, which was renamed as E+_1_.

### Phase 2: Pairing of the most efficient parental strains available and first generation progeny testing in the field

The most efficient *C*. *purpureum* parental strains were paired in Phase 2 (Table 2). Based on the subsequent field experiment, progenies R12_2_ and R15_2_ (later on designated as V1_2_ and V2_2_, respectively) had the lowest number of stump sprouts and *p*-values compared to controls. These strains were chosen for further pairings and investigation (see Table 2, Fig. 2a) although the final effect on sprout control can be expected only after several growing seasons [33]. Differences in the number of stump sprouts between the parental strains HY4, P4 and 3.11, and the control treatments were smaller (0.127 ≤ *p ≤* 0.892). Furthermore, the models revealed that the number of stump sprouts increased with an increase in basal diameter of a stump (*p* < 0.001).

### Phase 3: Pairing of the best progenies from Phases 1 and 2 and second generation progeny testing in the field

In Phase 3, strain E+_1_ from Phase 1 was further paired with V1_2_ and V2_2_ from Phase 2 (Table 2). The efficacy of the progenies in sprout control was further tested in the field. The best three progenies in this field experiment were R3_3_, R5_3_ and R9_3_ based on the lowest numbers of stump sprouts on investigated stumps and *p*-values compared to the controls (see Table 2, Fig. 2b). Differences in the number of stump sprouts between parental strains HY4 and 3.11 and the controls were smaller (*p* ≥ 0.590), but parental strain P4 was quite efficient in this experiment (*p* ≥ 0.110). Furthermore, the number of stump sprouts increased with an increase in basal diameter of a stump (*p* < 0.001).

### Phase 4: Long-lasting field experiment—final testing of the efficacy of the best progenies from Phases 2 and 3

Mortality

**Fig 3:**
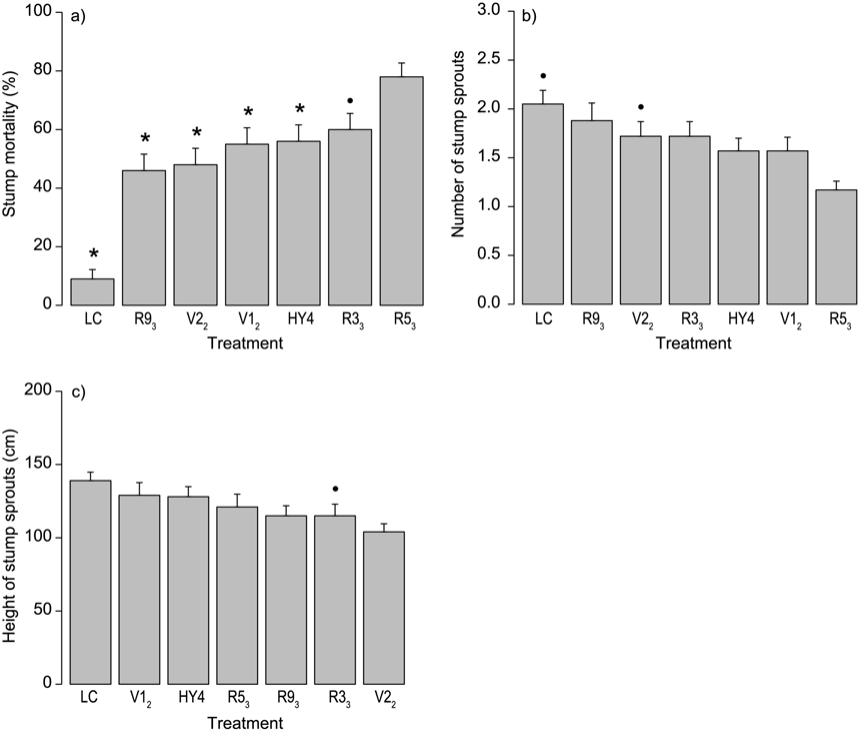
The Effects of Different Treatments on Birch Sprouting during Phase 4. The effects on a) the mortality of stump sprouts (*n* = 560), b) the number of stump sprouts in living stumps (*n* = 279), and c) the maximum height of stump sprouts in living stumps (*n* = 279) of birch (*Betula pendula* and *B*. *pubescens*) three growing seasons (autumn 2013) after the treatments. Means with standard errors are presented. Statistically significant differences between the *C*. *purpureum* strain R5_3_ and other treatments are indicated with an asterisk (*p* < 0.05) or a dot (0.05 ≤ *p* ≤ 0.10). See Tables 4–6.

**Fig 4:**
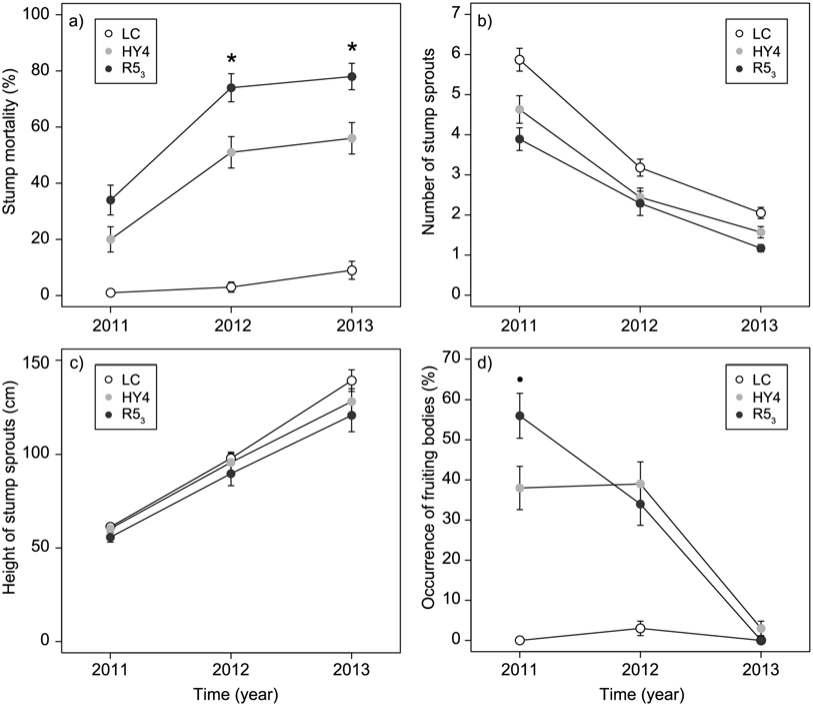
Comparison between the Best Progeny and Parent Strain of *C*. *purpureum*. The effects of *C*. *purpureum* strains R5_3_ (a progeny), HY4 (a parental strain), and the liquid control (cutting with inoculum medium without *C*. *purpureum*) on a) stump mortality (%), b) the number of stump sprouts (in living stumps), c) stump sprout height (in living stumps, cm), and d) the occurrence of fruiting bodies (%) on birch (*Betula pendula* and *B*. *pubescens*) stumps one, two and three growing seasons after the treatments (Phase 4). Means with standard errors are presented. Statistically significant differences (*p* < 0.05) between fungal strains R5_3_ and HY4 are indicated with an asterisk and indicative differences (0.05 ≤ *p* ≤ 0.10) with a dot. See Tables 4–7.

In the final field experiment (Phase 4), lasting three growing seasons (2011–2013), we found that one of the tested *C*. *purpureum* progenies, R5_3_, was considerably more efficient than other strains in the sprout control of birch. Mortality was clearly higher in stumps treated with R5_3_ especially two and three growing seasons after the treatments in 2012 and 2013 (Table 4). This was the only strain that differed statistically significantly from the parental strain HY4 three growing seasons (in 2013) after the treatments. After three growing seasons, the mortality in R5_3_ treated stumps was 78% whereas that for the best parental strain, HY4, was 56% and for the control 9% (cutting with inoculum medium without *C*. *purpureum*, see Table 4, Figs. 3a and 4a). The efficacy in sprout control was 28% lower for the parental strain HY4 than for R5_3_. Our results also revealed that another progeny isolate (R3_3_) was highly efficient in sprout control as 60% of the treated birch stumps were dead after three growing seasons. However, the efficacy for R3_3_ was 23% lower than for R5_3_. Furthermore, the mortality of stumps was higher in all the *C*. *purpureum* treatments than in the liquid control (0.001 *≤ p ≤* 0.099).

**Table 4:** Differences in Mortality of Birch Stumps between the *C*. *purpureum* Strain R5_3_ and Other Treatments.

Explanatory	Response variables					
variables	Mortality in 2011		Mortality in 2012		Mortality in 2013	
	*n* = 560		*n* = 559		*n* = 560	
	Coeff. ± SE	*p*	Coeff. ± SE	*p*	Coeff. ± SE	*p*
Intercept	-2.001 ± 0.656	**0.002**	-0.196 ± 0.465	0.673	0.621 ± 0.528	0.239
Treatment						
-LC^a^	-4.133 ± 1.199	**0.001**	-4.952 ± 0.770	**<0.001**	-3.891 ± 0.543	**<0.001**
-HY4^b^	-0.639 ± 0.533	0.231	-0.978 ± 0.353	**0.006**	-1.030 ± 0.413	**0.013**
-R12_2_ = V1_2_ ^c^	-0.538 ± 0.529	0.309	-1.344 ± 0.358	**<0.001**	-1.151 ± 0.412	**0.005**
-R15_2_ = V2_2_ ^c^	-2.070 ± 0.624	**0.001**	-1.763 ± 0.362	**<0.001**	-1.596 ± 0.414	**<0.001**
-R3_3_ ^d^	-0.397 ± 0.513	0.439	-0.833 ± 0.347	**0.016**	-0.763 ± 0.412	0.064
-R9_3_ ^d^	-1.395 ± 0.570	**0.014**	-1.662 ± 0.358	**<0.001**	-1.456 ± 0.412	**<0.001**
Stump basal diameter (mm)	0.112 ± 0.038	**0.004**	0.077 ± 0.030	**0.010**	0.022 ± 0.030	0.462
Saplings around	-0.040 ± 0.032	0.207	0.017 ± 0.020	0.412	0.024 ± 0.023	0.303
Tree volume (m^3^ ha^-1^)	0.038 ± 0.008	**<0.001**	0.027 ± 0.006	**<0.001**	0.020 ± 0.007	**0.005**

The effect of different treatments (*C*. *purpureum* strain or liquid control (LC)), the basal area of investigated stumps, the number of saplings and the volume of trees around an investigated sapling on the mortality of birch (*Betula pendula* and *B*. *pubescens*) stumps in eight regeneration areas of spruce (*Picea abies*) three months (in 2011), one year (in 2012) and two years (in 2013) after the treatments (generalized linear mixed model results). All stumps have been included in the models. Statistically significant *p*-values (*p* < 0.05) for the model coefficients are in bold and indicative results have been underlined (0.05 ≤ *p* ≤ 0.10). The sign of a coefficient indicates whether an explanatory variable has an increasing (+) or decreasing (-) effect on the mortality of stumps. See Figs. 3a and 4a. Note: the subscript of a *C*. *purpureum* progeny relates to the breeding phase (see Table 2).

^a^ Liquid control i.e., cut stumps were spread with inoculum medium without *C*. *purpureum*.

^b^ The best parent stain based on an earlier study [13].

^c^ The best progenies from Phase 2.

^d^ The best progenies from Phase 3.

### Sprout numbers

Our results showed that the number of stump sprouts (in the living birches) was lower for treatment R5_3_ than for treatments V1_2_, V2_2_, and the liquid control in 2011 (see Table 5). After three growing seasons (in 2013), the number of sprouts in living stumps was still the lowest in stumps treated with R5_3_ but the differences were not significant any more (see Table 5, Fig. 3b). The number of stump sprouts in living stumps was lower in all the *C*. *purpureum* treatments compared to the liquid control (0.001 ≤ *p* ≤ 0.085).

**Table 5:** Differences in the Number of Stump Sprouts of Living Birches between the *C*. *purpureum* Strain R5_3_ and Other Treatments.

Explanatory	Response variables					
variables	Number of stump sprouts in 2011		Number of stump sprouts in 2012		Number of stump sprouts in 2013	
	*n* = 454		*n* = 317		*n* = 279	
	Coeff. ± SE	*p*	Coeff. ± SE	*p*	Coeff. ± SE	*p*
Intercept	1.044 ± 0.121	**0.001**	0.756 ± 0.232	**0.001**	0.170 ± 0.286	0.552
Treatment						
-LC^a^	0.343 ± 0.099	**0.001**	0.276 ± 0.160	0.085	0.448 ± 0.238	0.060
-HY4^b^	0.155 ± 0.105	0.139	0.105 ± 0.178	0.554	0.209 ± 0.258	0.418
-R12_2_ = V1_2_ ^c^	0.272 ± 0.105	**0.010**	-0.002 ± 0.177	0.991	0.240 ± 0.257	0.350
-R15_2_ = V2_2_ ^c^	0.271 ± 0.102	**0.008**	0.212 ± 0.172	0.218	0.415 ± 0.249	0.095
-R3_3_ ^d^	0.091 ± 0.107	0.391	-0.126 ± 0.183	0.494	0.251 ± 0.261	0.336
-R9_3_ ^d^	0.130 ± 0.104	0.210	0.030 ± 0.171	0.859	0.334 ± 0.251	0.184
Stump basal diameter (mm)	0.044 ± 0.007	**<0.001**	0.029 ± 0.011	**0.009**	0.024 ± 0.014	0.076
Saplings around	-0.008 ± 0.005	0.141	-0.021± 0.009	**0.020**	-0.015 ± 0.010	0.147
Tree volume (m^3^ ha^-1^)	-0.006 ± 0.002	**<0.001**	-0.005 ± 0.004	0.143	-0.005 ± 0.003	0.125

The effect of different treatments (*C*. *purpureum* strain or liquid control (LC)), the basal area of investigated stumps, the number of saplings and the volume of trees around an investigated sapling on the number of stump sprouts of birch (*Betula pendula* and *B*. *pubescens*) in eight regeneration areas of spruce (*Picea abies*) three months (in 2011), one year (in 2012) and two years (in 2013) after the treatments (generalized linear mixed model results). Living stumps have been included in the models. Statistically significant *p*-values (*p* < 0.05) for the model coefficients are in bold and indicative results have been underlined (0.05 ≤ *p* ≤ 0.10). The sign of a coefficient indicates whether an explanatory variable has an increasing (+) or decreasing (-) effect on the number of stump sprouts. See Figs. 3b and 4b. Note: the subscript of a *C*. *purpureum* progeny relates to the breeding phase (see Table 2).

^a^ Liquid control i.e., cut stumps were spread with inoculum medium without *C*. *purpureum*.

^b^ The best parent stain based on an earlier study [13].

^c^ The best progenies from Phase 2.

^d^ The best progenies from Phase 3.

### Sprout height

Treatment R5_3_ was not better than the other *C*. *purpureum* treatments in terms of the maximum height of stump sprouts (see Table 6, Fig. 3c). In fact, after the third growing season (in 2013), sprout height was indicatively lower in stumps treated with the progeny R3_3_ than with R5_3_. However, the maximum height of stump sprouts in living stumps was lower in all *C*. *purpureum* treatments than in the liquid control (0.005 ≤ *p* ≤ 0.096).

**Table 6:** Differences in the Height of Stump Sprouts of Living Birches between the *C*. *purpureum* Strain R5_3_ and Other Treatments.

Explanatory	Response variables					
variables	Stump sprout height in 2011		Stump sprout height in 2012		Stump sprout height in 2013	
	*n* = 454		*n* = 317		*n* = 279	
	Coeff. ± SE	*p*	Coeff. ± SE	*P*	Coeff. ± SE	*p*
Intercept	46.065 ± 4.588	**<0.001**	75.190 ± 10.645	**<0.001**	112.429 ± 16.939	**<0.001**
Treatment						
-LC^a^	4.330 ± 3.557	0.230	3.155 ± 7.444	0.674	5.965 ± 11.204	0.598
-HY4^b^	-1.704 ± 3.620	0.640	2.906 ± 7.900	0.715	-5.446 ± 11.974	0.652
-R12_2_ = V1_2_ ^c^	0.717 ± 3.668	0.846	-0.057 ± 7.785	0.994	1.679 ± 11.857	0.888
-R15_2_ = V2_2_ ^c^	0.766 ± 3.580	0.832	-9.614 ± 7.727	0.221	-9.347 ± 11.767	0.432
-R3_3_ ^d^	-2.646 ± 3.692	0.478	-8.554 ± 8.040	0.294	-21.540 ± 12.295	0.088
-R9_3_ ^d^	2.173 ± 3.644	0.554	-7.848 ± 7.736	0.317	-15.882 ± 11.896	0.190
Stump basal diameter (mm)	1.466 ± 0.205	**<0.001**	2.403 ± 0.454	**<0.001**	3.598 ± 0.725	**<0.001**
Saplings around	-0.180 ± 0.180	0.316	-0.718 ± 0.396	0.071	-1.692 ± 0.611	**0.006**
Tree volume (m^3^ ha^-1^)	-0.163 ± 0.071	**0.027**	-0.153 ± 0.156	0.335	-0.553 ± 0.229	**0.021**
Browsing	-6.459 ± 1.649	**<0.001**	1.263 ± 3.413	0.712	3.365 ± 6.634	0.613

The effect of different treatments (*C*. *purpureum* strain or liquid control (LC)), the basal area of investigated stumps, the number of saplings and the volume of trees around an investigated sapling, and browsing on the maximum height of stump sprouts of birch (*Betula pendula* and *B*. *pubescens*) in eight regeneration areas of spruce (*Picea abies*) three months (in 2011), one year (in 2012) and two years (in 2013) after the treatments (linear mixed model results). Living stumps have been included in the models. Statistically significant *p*-values (*p* < 0.05) for the model coefficients are in bold and indicative results have been underlined (0.05 ≤ *p* ≤ 0.10). The sign of a coefficient indicates whether an explanatory variable has an increasing (+) or decreasing (-) effect on the maximum height of stump sprouts. See Figs. 3c and 4c. Note: the subscript of a *C*. *purpureum* progeny relates to the breeding phase (see Table 2).

^a^ Liquid control i.e., cut stumps were spread with inoculum medium without *C*. *purpureum*.

^b^ The best parent stain based on an earlier study [13].

^c^ The best progenies from Phase 2.

^d^ The best progenies from Phase 3.

### Fruiting schedule

In 2011, stumps treated with treatment R5_3_ had higher occurrence of fruiting bodies (56%) than stumps treated with HY4 (38%), V1_1_ (39%), V2_1_ (41%), R3_3_ (46%) and R9_3_ (48%). However, only some statistically indicative differences between the strains were found (see Table 7). In 2012, positive coefficients for the other *C*. *purpureum* treatments indicated that the occurrence of fruiting bodies was lower in the R5_3_ treatment. In 2013, no differences in the occurrence of fruiting bodies were found. The occurrence of fruiting bodies on stumps treated with *C*. *purpureum* decreased with time, ca. 45% of stumps had fruiting bodies in 2011, whereas later on in 2012 and 2013, 39% and 4% of stumps had fruiting bodies, respectively. Only two stumps in the control treatment had fruiting bodies in 2012 (see Fig. 4d).

**Table 7:** Differences in the Occurrence of Fruiting Bodies in Birch Stumps between the *C*. *purpureum* Strain R5_3_ and Other Treatments.

Explanatory	Response variables					
variables	Occurrence of fruiting bodies in 2011		Occurrence of fruiting bodies in 2012		Occurrence of fruiting bodies in 2013	
	*n* = 480		*n* = 479		*n* = 480	
	Coeff. ± SE	*p*	Coeff. ± SE	*p*	Coeff. ± SE	*p*
Intercept	-2.339 ± 0.752	**0.002**	-2.382 ± 0.570	**<0.001**	-	-
Treatment						
-HY4^a^	-0.970 ± 0.496	0.051	0.248 ± 0.350	0.480	-	-
-R12_2_ = V1_2_ ^b^	-0.959 ± 0.511	0.061	0.022 ± 0.350	0.950	-	-
-R15_2_ = V2_2_ ^b^	-0.698 ± 0.490	0.154	0.759 ± 0.346	**0.028**	-	-
-R3_3_ ^c^	-0.678 ± 0.489	0.166	0.031 ± 0.355	0.931	-	-
-R9_3_ ^c^	-0.584 ± 0.481	0.225	0.209 ± 0.349	0.549	-	-
Stump basal diameter (mm)	0.258 ± 0.040	**<0.001**	0.145 ± 0.032	**<0.001**	-	-
Saplings around	-0.014 ± 0.031	0.640	0.007 ± 0.023	0.752	-	-
Tree volume (m^3^ ha^-1^)	0.012 ± 0.010	0.249	0.001 ± 0.007	0.910	-	-

The effect of different treatments (*C*. *purpureum* strain), the basal area of investigated stumps, the number of saplings and the volume of trees around an investigated sapling on the probability of occurrence of fruiting bodies on birch (*Betula pendula* and *B*. *pubescens*) stumps in eight regeneration areas of spruce (*Picea abies*) three months (in 2011) and one year (in 2012) after the treatments (generalized linear mixed model results). The model for year 2013 was not estimated because the occurrence of fruiting bodies was too low (4%). All stumps except those in the liquid control have been included in the models. Statistically significant *p*-values (*p* < 0.05) for the model coefficients are in bold and indicative results have been underlined (0.05 ≤ *p* ≤ 0.10). The sign of a coefficient indicates whether an explanatory variable has an increasing (+) or decreasing (-) effect on the occurrence of fruiting bodies. See Fig. 4d. Note: the subscript of a *C*. *purpureum* progeny relates to the breeding phase (see Table 2).

^a^ The best parent stain based on an earlier study [13].

^b^ The best progenies from Phase 2.

^c^ The best progenies from Phase 3.

### Effects of stump sizes and environmental factors

One and two growing seasons after the treatments, an increase in stump basal diameter increased stump mortality (see Table 4). Furthermore, an increase in the volume of retention trees increased mortality during the study. Three growing seasons after the treatments, an increase in the basal diameter of stumps increased the number and maximum height of stump sprouts in living stumps, whereas an increase in the number of surrounding saplings and the volume of retention trees decreased the maximum height (see Tables 5 and 6). Browsing decreased the maximum height in 2011. The probability of occurrence of fruiting bodies increased with an increase in stump diameter (see Table 7).

### Differences between *Betula pendula* and *B*. *pubescens*

Our results revealed that only slight differences were found between the two birch species. Mortality of *B*. *pendula* (58% of investigated stumps) was indicatively higher than for *B*. *pubescens* (48%, *p* = 0.097). However, no differences were found when the number and maximum height of stump sprouts were compared (*p* = 0.574 and *p* = 0.707, respectively).

## Discussion

Our results showed that breeding can be used to increase the efficacy of *C*. *purpureum* as a biocontrol agent. After the whole breeding process, *C*. *purpureum* strain R5_3_ was statistically significantly better than the best original parental strain HY4 and subsequent parental strain V1_2_ created during the process. After three growing seasons, the mortality of birch stumps ca. 1 cm in diameter treated with R5_3_ was 78% whereas those with parental strains HY4 and V1_2_ were only 56% and 55%, respectively. Thus, it seems that our breeding process was successful, supporting our initial hypothesis.

The efficacy of HY4 has been investigated in an earlier study with birch stumps ca. 3–4 cm in diameter resulting in more than 90% mortality after two growing seasons [13]. The lower mortalities observed in the present study was expected as it is known that mortality is usually lower for smaller stumps [9]. Therefore, the mortality caused by the best strain from our breeding program (78%) in small stumps with a diameter of about 1 cm can be considered a promising result because in regeneration areas of spruce (*Picea abies*) and pine (*Pinus sylvestris*), non-crop species of this size are usually removed. Thus, high biocontrol efficacy with *C*. *purpureum* can provide one option to lower costs for sprout control via decreasing the number of repeated cuttings (see [34]). Sprout control also allows better growth conditions for more valuable conifers because competition with broad-leaved trees can reduce their growth and even induce mortality [3,34,35].

It seems that *C*. *purpureum* is especially efficient in the sprout control of birch, as for yellow birch (*Betula alleghaniensis* Britt.) at least 96% of inoculated stumps died within one year [8], similarly as with silver and downy birch after three growing seasons [33], whereas in the study of Roy et al. [14], the mortality of paper birch (*Betula papyrifera* Marsh.) was ca. 75% after four growing seasons. Also, fruiting bodies have been especially abundant on inoculated yellow (87% of the treated stumps) and paper birch stumps (100%) [8,36].

In our study, after the first growing season in 2011, the occurrence of fruiting bodies was highest (56%) with strain R5_3_ indicating that this fungus was able to penetrate wood faster than the parental strain HY4 and the other *C*. *purpureum* strains. Investigations with red alder (*Alnus rubra* Bong.) have revealed a close relationship between the time of mortality and the occurrence of fruiting bodies: peaks in fruiting body formation occur one year before, the same year or one year after mortality, and those trees that died slowly supported fruiting bodies for a longer time [36].

Although significant differences were not found in the number of stump sprouts per living stump between the progeny strain R5_3_ and the parental strain HY4, the treatment with R5_3_ resulted in a lower number of sprouts in living stumps (see Figs. 3b and 4b). Furthermore, results relating to the progeny strain R5_3_ indicate that the number of sprouts per stump was lower than in the control in every investigated year. However, in terms of the maximum height of sprouts (in living stumps), hardly any differences between the treatments were found (see also [13]).

Our results revealed that an increasing volume of trees around an investigated stump increased stump mortality. This is in accordance with earlier findings as shading of neighboring trees has been shown to have a profound effect on shoot growth due to a greater proportion of shoot buds dying under heavy shading [37,38]. Furthermore, our results showed that an increase in the number of surrounding saplings decreased the number and maximum height of stump sprouts (see also [39]). On the other hand, high mortality with *C*. *purpureum* inoculated stumps (especially with R5_3_) may provide more growing space for those stumps that are still living. This may be the reason why significant differences between the treatments were not observed in stump numbers and height.

All *C*. *purpureum* strains investigated were originally collected from birch stumps in southern and middle Finland (see Methods section and Table 1). However, investigations from Finland, Canada and New Zealand showed that *C*. *purpureum* strains are genetically diverse, and are not associated with a specific host species or ecological region [11,17,18]. Thus, it is possible that the most efficient strain, R5_3_, is efficient also against the sprouting of other tree species and in other geographical areas.

Furthermore, earlier studies have indicated that a single genotype can be used as inoculum without the risk of introducing genes that differ significantly from local populations [11,17]. The best strain R5_3_ was developed by traditional breeding (via consequent pairings of selected mycelia), i.e., similarly like any other *C*. *purpureum* strain in the nature. In that sense this strain does not differ from its natural counterparts. Moreover, as the fungus has no asexual stage, the genetic combination of a single exceptionally efficient biocontrol fungus breaks up in meiosis before the spores are released. Thus, the same *C*. *purpureum* genotype applied on cut stumps cannot spread further in forest ecosystems, and thus there is no risk for explosion of the biocontrol strain.

Classical breeding experiments with biocontrolling fungi are scarce. However, in addition to ours, Wall et al. [10] compared the efficacy of *C*. *purpureum* monokaryons and their dikaryon progenies in causing wood tissue mortality, but could not find any differences. However, their experiment was not tested under field conditions. Another biocontrol fungus, *Phlebiopsis gigantea*, was bred to improve the efficacy against *Heterobasidion* root rot in forests [27]. Results of that study indicated that progeny strains had better properties against *H*. *parviporum* than the parental strains. Furthermore, classical pairings were successfully used to improve temperature tolerance of fermenting yeasts using a backcross approach [25]. Thus, these studies (including the present one) show that improving by breeding may help in developing more efficient fungal strains for different purposes.

However, breeding studies with field experiments may be time consuming as seen in our study. Time for follow-up is usually limited, which forces us to choose the best strains for subsequent steps after a short time period. For example, in our study, we had to choose progeny isolates for the next pairings after the first growing season (both in 2009 and 2010) based only on the number of stump sprouts in different *C*. *purpureum* treatments (a low mortality did not allow for mortality analyses). Moreover, as seen also in the present study, the efficacy of *C*. *purpureum* is not tightly associated with the parents, because each parent can produce relatively virulent as well as avirulent strains [10]. This is not surprising as Wall et al. [10] have stated that the inheritance of efficacy is probably multifactorial and complex and may be subject to different modifying factors under field and laboratory conditions. Thus, final efficacy should always be investigated in field experiments. This view was supported by our results as the best strain in terms of laccase activity in the laboratory, E+_1_, was not efficient in sprout control (see Fig. 2b) although some of its progenies (R5_3_ and R3_3_) in the end showed the best ability to prevent sprouting of broad-leaved trees in the field.

## Conclusions

We showed that traditional breeding can increase the efficacy of *C*. *purpureum* in the sprout control of birch. Mortality of the treated birch stumps was highest when the progeny strain R5_3_ was used to control sprouting. This resulted in 78% mortality in birch stumps 1 cm in diameter. The efficacy of strain R5_3_ was significantly higher than that of the investigated parental strain HY4 (56%). The effect of sprout control was especially pronounced when the volume of trees and the number of surrounding saplings was high. However, breeding experiments that aim at increasing the efficacy of sprout control are time consuming because efficacy has to be investigated in long-lasting field experiments.
